# Combined Mesenchymal Stem Cells and Low-Energy Shock Wave Therapy Significantly Reduced Inflammation, Oxidative Stress and Preserved Podocytes in Diabetic Nephropathy

**DOI:** 10.3390/life16091550

**Published:** 2026-09-16

**Authors:** Chang-Chun Hsiao, Hsien-Wei Hsiao, Yu-Hsuan Liu, Chien-Te Lee

**Affiliations:** 1 Division of Nephrology, Department of Internal Medicine, Kaohsiung Municipal Fong-Shan Hospital and Chang Gung University College of Medicine, Taoyuan 33382, Taiwan; cchsiao@mail.cgu.edu.tw (C.-C.H.); 498h0007@stust.edu.tw (Y.-H.L.); 2 Graduate Institute of Clinical Medical Sciences, Chang Gung University College of Medicine, Taoyuan 33382, Taiwan; 3 Department of Ophthalmology, Kaohsiung Chang Gung Memorial Hospital, Kaohsiung 83325, Taiwan; b04401034@ntu.edu.tw

**Keywords:** low-energy shock wave, Wharton’s jelly mesenchymal stem cells, diabetic nephropathy, podocyte regeneration

## Abstract

Diabetic nephropathy (DN) is among the leading microvascular complications of diabetes mellitus and a major cause of end-stage renal disease. Current treatments slow disease progression but do not restore lost podocytes. We tested whether Wharton’s jelly-derived mesenchymal stem cells (WJ-MSCs) combined with low-energy shock wave (SW) therapy could be a safe and effective therapy in a streptozotocin-induced rat model of DN. Diabetic rats received 107 WJ-MSCs together with SW (0.13 mJ/mm^2^, 200 shocks) once a week for five weeks. The combined regimen lowered urinary microalbumin and the albumin-to-creatinine ratio, attenuated glomerular hypertrophy, and reduced fibronectin and collagen I deposition. Podocyte markers (WT-1, synaptopodin, nephrin) recovered toward normal levels. WJ + SW therapy suppressed NF-κB and IL-6 while raising IL-4 and IL-10, lowered NOX4 expression, and increased the antioxidant enzyme HO-1. Renal macrophages also shifted from the pro-inflammatory M1 phenotype to the reparative M2 phenotype. Together, these findings indicate that combining WJ-MSCs with low-energy SW therapy acts on multiple pathogenic axes of DN and may offer a potential interventional strategy for this inescapable and progressive complication.

## 1. Introduction

Diabetic nephropathy (DN) remains a leading cause of end-stage renal disease worldwide, and its global burden has risen steadily over the past three decades in parallel with the type 2 diabetes epidemic [1,2]. Glycemic control, renin–angiotensin system blockade, and newer agents such as SGLT2 inhibitors [3] and finerenone [4] slow disease progression, yet none of these therapies preserve podocytes or reverse established glomerular injury. A regenerative strategy is therefore urgently needed [5].

DN is driven by a complex interplay of hyperglycemia, chronic inflammation, oxidative stress, and apoptosis [5,6]. IL-6, TNF-α, and NF-κB are central drivers of renal inflammation and fibrosis [6,7], while reactive oxygen species produced by NADPH oxidases—chiefly NOX4 in the renal cortex—amplify inflammation and promote podocyte loss and matrix accumulation [8,9]. Clinically, these processes manifest as podocyte injury, glomerular hypertrophy, fibrosis, and progressive proteinuria [5,10].

Podocytes are terminally differentiated cells that hold the glomerular filtration barrier together; their loss leads directly to proteinuria and glomerulosclerosis, which makes preserving and replacing them a central goal in DN treatment [10,11]. Nephrin, a key slit-diaphragm protein, is required for podocyte function and is increasingly used as an early biomarker of DN [11]. Parietal epithelial cells of the glomerulus appear to act as a local progenitor pool capable of replenishing damaged podocytes [12,13]. At the same time, a skewed macrophage profile—M1 dominant, M2 deficient—drives tubulointerstitial inflammation and fibrosis in the diabetic kidney [14,15].

Wharton’s jelly-derived mesenchymal stem cells (WJ-MSCs), obtained from the umbilical cord, carry the canonical MSC markers CD29, CD44, CD73, CD90, and CD105 but lack hematopoietic markers such as CD14, CD31, CD34, and CD133 [16,17]. Their low MHC class II expression and limited costimulatory activity give them immune-privileged status, allowing them to dampen T-, B-, and NK-cell proliferation and to favor a regulatory T-cell milieu [18,19]. Most of their therapeutic effect is paracrine: secreted hepatocyte growth factor (HGF), epidermal growth factor (EGF), vascular endothelial growth factor (VEGF), and immunomodulatory cytokines blunt inflammation, suppress apoptosis, and lower oxidative stress [20,21,22]. Preclinical and early clinical data have begun to show that WJ-MSCs, and even their conditioned media alone, protect the kidney in diabetic and ischemic injury models [23,24].

Low-energy extracorporeal shock wave (SW) therapy is a non-invasive treatment that delivers mechanical signals to tissue and triggers cellular responses through mechanotransduction [15,25]. In diabetic kidneys, pancreatic beta cells, and wound-healing models, SW therapy has been shown to dampen oxidative stress, inflammation, and apoptosis while stimulating proliferation, angiogenesis, and repair [15,25,26]. A recent systematic review of low-intensity ESWT in renal disease concluded that the treatment reduces inflammation, fibrosis, and capillary rarefaction without renal toxicity in both animal and human studies [27]. SW therapy can also push macrophages from the M1 toward the M2 phenotype, which helps to quiet chronic renal inflammation [15,25].

Because the two modalities act through different but complementary mechanisms—WJ-MSCs through paracrine and immunomodulatory effects, SW therapy through mechanotransduction and tissue repair—we reasoned that combining them might cover more of the pathological landscape of DN. In this study, we treated streptozotocin-induced diabetic rats with WJ-MSCs plus low-energy SW (WJ + SW) and asked whether the combination would reduce inflammation, oxidative stress, and apoptosis, restore nephrin, and lower microalbuminuria. We also examined changes in renal architecture, macrophage polarization, and redox balance.

## 2. Materials and Methods

### 2.1. Animals

Male Wistar rats (age: 6–7 weeks, 250–300 g) were purchased from BioLASCO (Taipei, Taiwan). The animal center at Kaohsiung Chang Gung Memorial Hospital provided veterinary care to the Wistar rats. All animals were housed at 22–24 °C under a 12 h light/dark cycle. The rats were given tap water and standard chow with the following composition: 19.2% crude protein, 4.1% crude fat, 6.1% crude fiber, 11.3% moisture, 5.9% ash content, 0.7% calcium, and 0.8% phosphorus (Altromin 1324, Altromin Spezialfutter GmbH & Co. KG Im Seelenkamp 20, 32791 Lage, Germany). Rats were randomly assigned to the diabetic nephropathy (DN) group (*N* = 8), the WJ-MSC therapy combined with low-energy SW therapy (WJ + SW) group (*N* = 8), or the normal group (*N* = 8). This study was approved by the Institutional Animal Care and Use Committee (IACUC: 2021091703) at Kaohsiung Chang Gung Memorial Hospital.

### 2.2. Establishment of a Diabetic Nephropathy Rat Model

A diabetic nephropathy rat model was established according to our previously published protocol [28]. DN was induced by intraperitoneal injection of streptozotocin (50 mg/kg, Sigma-Aldrich, St. Louis, MO, USA) [28]. Blood glucose was maintained at ~350 mg/dL via insulin treatment (Humulin 70/30, regular insulin 30%, Eli Lilly, Indianapolis, IN, USA) (0.4 unit/rat).

### 2.3. Flow Cytometry

WJ-MSCs (Cellular Engineering Technologies Inc., Coralville, IA, USA) were detached using 0.05% trypsin–EDTA in PBS (1×, pH 7.4; Gibco, New York, NY, USA), incubated with the respective antibodies, and conjugated with fluorescein isothiocyanate or phycoerythrin against the following markers: CD29, CD44, CD73, CD90, CD105, CD166, CD14, CD31, CD34, CD133, and CD117. Cells were analyzed using a flow cytometer (BD LSRII, Franklin Lakes, NJ, USA).

### 2.4. Low-Energy Shock Wave Therapy Combined with WJ Therapy (WJ + SW)

WJ-MSCs were cultured in MSC Expansion Media and harvested for use after five to six passages. Six weeks after diabetes was successfully induced, 10^7^ WJ-MSCs in 250 μL hyaluronic acid (CAS 9067-32-7, Merck Ltd., Taipei, Taiwan), combined with Hank’s balanced salt solution (H8264, Merck Ltd., Taipei, Taiwan), was administered into the kidney via subcapsular injection for each rat. Low-energy SW therapy was performed using ultrasonics (Toshiba, Tokyo, Japan); ultrasonic gel was applied to each rat’s abdomen after shaving the fur. The SW intensity was 0.13 mJ/mm^2^ with 200 shocks delivered at 200 pulses per minute. A shock wave applicator (EvoTron R05, High Medical Technologies, Lengwil, Switzerland) probe was placed on the abdominal wall over the kidneys. Each animal received once-weekly treatment for 5 weeks. Details of the treatment protocol were shown in our previously published methods [15,25,28].

### 2.5. Measurement of Creatinine and Microalbumin

Creatinine and microalbumin were measured according to our previously published protocol [28]. Creatinine was measured using a microplate assay kit (Abcam, Cambridge, UK), and microalbumin was determined using a microalbumin ELISA kit (Abcam, Cambridge, UK). Glycated hemoglobin (HbA1C) was measured utilizing Roche, cobas^®^ HbA1c Test Gen. 2 (Basel, Switzerland).

### 2.6. Measurements of IL-6, IL-4, IL-10, HGF, EGF and Nephrin

Quantikine ELISA kits were used to determine renal tissue expression of IL-6, IL-4, and IL-10 and blood levels of HGF and EGF according to the manufacturer’s instructions (R&D Systems, Minneapolis, MN, USA). Urine nephrin levels were determined by Elabscience (Houston, TX, USA).

### 2.7. Real-Time Quantitative PCR Analysis

Real-time quantitative PCR was performed using our previously published protocols [15,25,26]. Gene expression was quantified using SYBR Green Master Mix on the 7500 Real-time PCR System (Applied Biosystems, Foster City, CA, USA).

### 2.8. Hematoxylin and Eosin (HE) Staining

Kidney tissues were fixed with 4% paraformaldehyde and embedded in paraffin. After deparaffinization and rehydration, the paraffin sections (3 μm) were stained with hematoxylin and eosin according to the manufacturer’s instructions. The glomerular volume was calculated as in our previous publications [15,25].

### 2.9. Immunofluorescence (IF)

Immunofluorescence staining was performed using our previously published protocols [15,25]. Kidney tissue slides were probed with a primary antibody against synaptopodin and heme oxygenase-1 (HO-1) (Abcam, Cambridge, UK). Ten glomeruli per stained slide were randomly selected for observation using an Olympus confocal microscope (Olympus FluoView 1000, Olympus Co., Tokyo, Japan). Positively immunolabeled cells and total cells per high-power field were counted in each section.

### 2.10. Western Blot

Western blotting was performed according to our previously published protocols [15,25,26]. Protein samples (30 μg) were separated by SDS-PAGE, transferred to polyvinylidene fluoride membranes (Millipore, Burlington, MA, USA), and probed with primary antibodies against β-actin (Sigma, St. Louis, MO, USA), WT-1 (Abcam Cambridge, UK), cleaved caspase-3 (Cell Signaling, Danvers, MA, USA), NF-κB (Abcam), CD68 (Abcam), NOX4 (Abcam), and HO-1 (Abcam) at 4 °C overnight, followed by horseradish peroxidase-conjugated secondary IgG.

### 2.11. Immunohistochemistry (IHC)

IHC analysis was performed according to our previously published protocols [15,25,26]. Renal tissue sections were incubated with primary antibodies against fibronectin, collagen I, PCNA, CD68 and NOX4 (all from Abcam, Cambridge, UK) diluted in blocking solution overnight at 4 °C, then probed with secondary antibody (Vector Laboratories, Burlingame, CA, USA) for 1 h at room temperature. Ten slides per rat were randomly selected for observation using an Axioskop 2 plus microscope (Carl Zeiss, Oberkochen, Germany). Three random images from each region were taken and analyzed using image analysis software (Image-Pro Plus 6.0, Media Cybernetics, Silver Spring, MD, USA).

### 2.12. Statistical Analysis

All experiments were repeated three to four times. Statistical analyses were performed using SPSS (version 13.0, SPSS, Chicago, IL, USA). Data are presented as mean ± SEM. Statistical significance between two groups was determined using ANOVA. A *p* < 0.05 was considered statistically significant.

## 3. Results

### 3.1. WJ-MSC Surface Marker Expression

**Figure 1:**
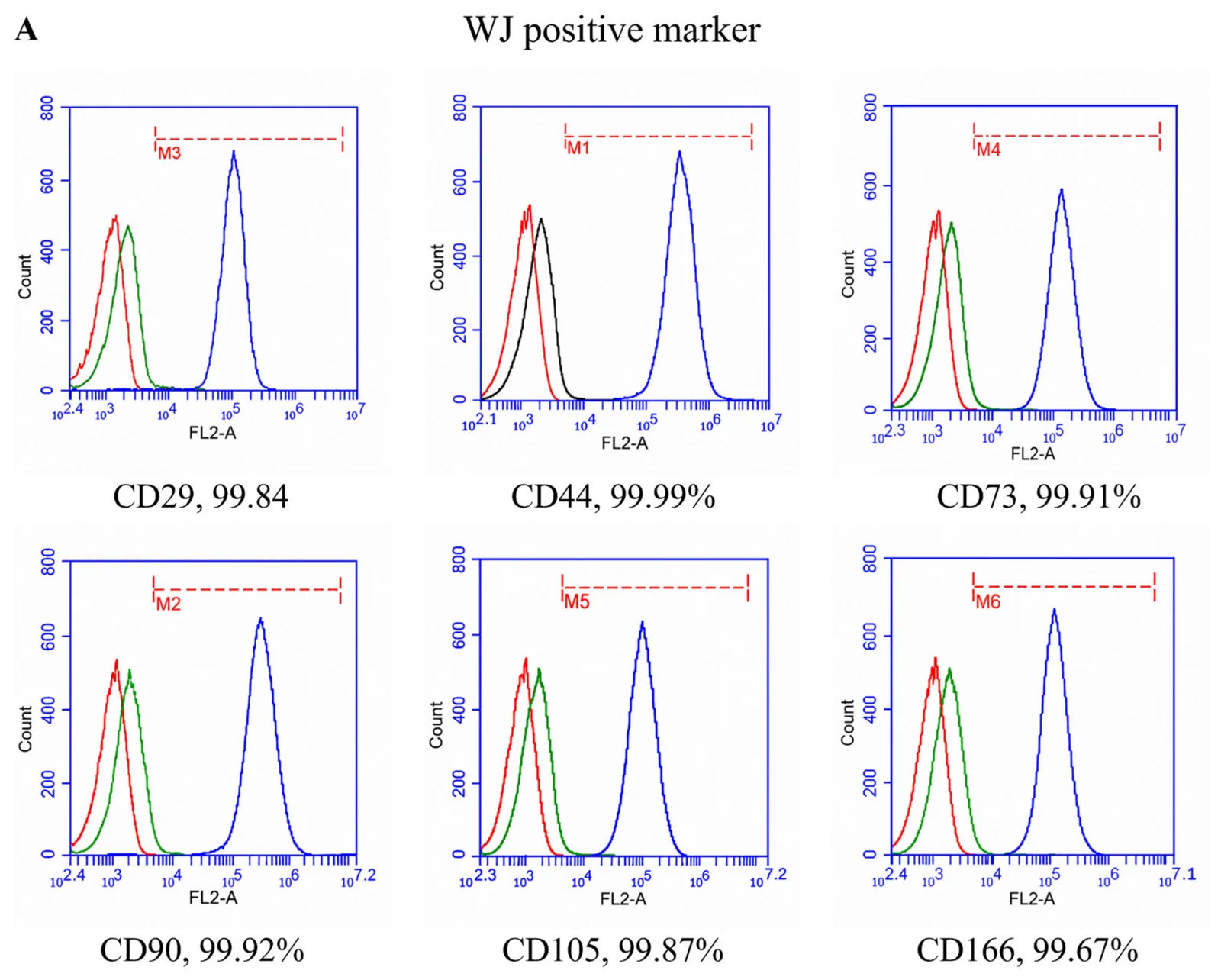

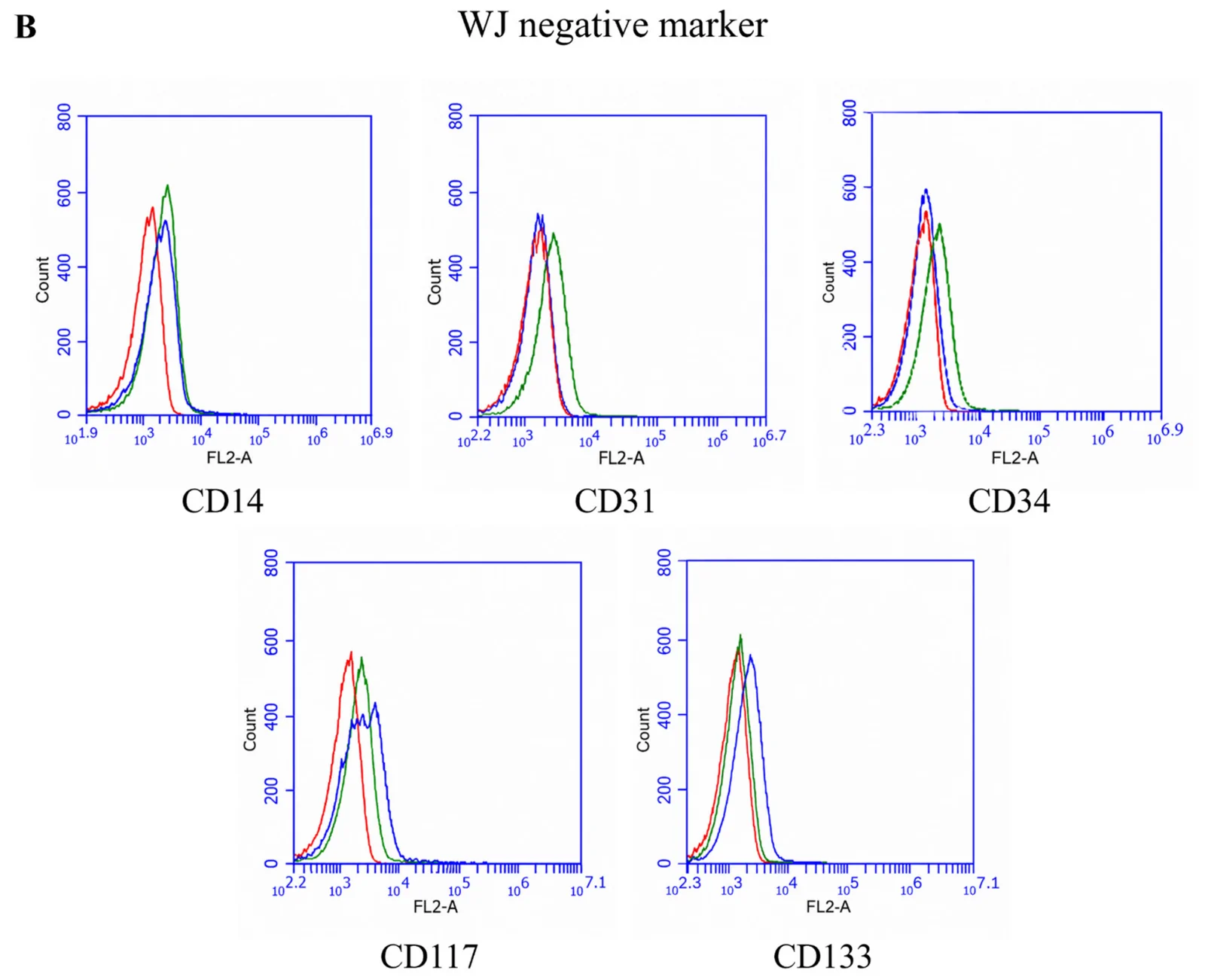
WJ-MSC surface marker expression analyzed by flow cytometry. (**A**) WJ-positive surface markers; (**B**) WJ-negative surface markers.

Before initiating therapy, we confirmed the WJ-MSC phenotype by flow cytometry. The cells were uniformly positive for the mesenchymal markers CD29 (99.84%), CD44 (99.99%), CD73 (99.91%), CD90 (99.92%), CD105 (99.87%), and CD166 (99.67%) (Figure 1A) and negative for the hematopoietic markers CD14, CD31, CD34, CD117, and CD133 (Figure 1B), consistent with previously published WJ-MSC characterization [16,17].

### 3.2. Laboratory Data and Glomerular Hypertrophy Before and After WJ + SW Therapy

**Figure 2:**
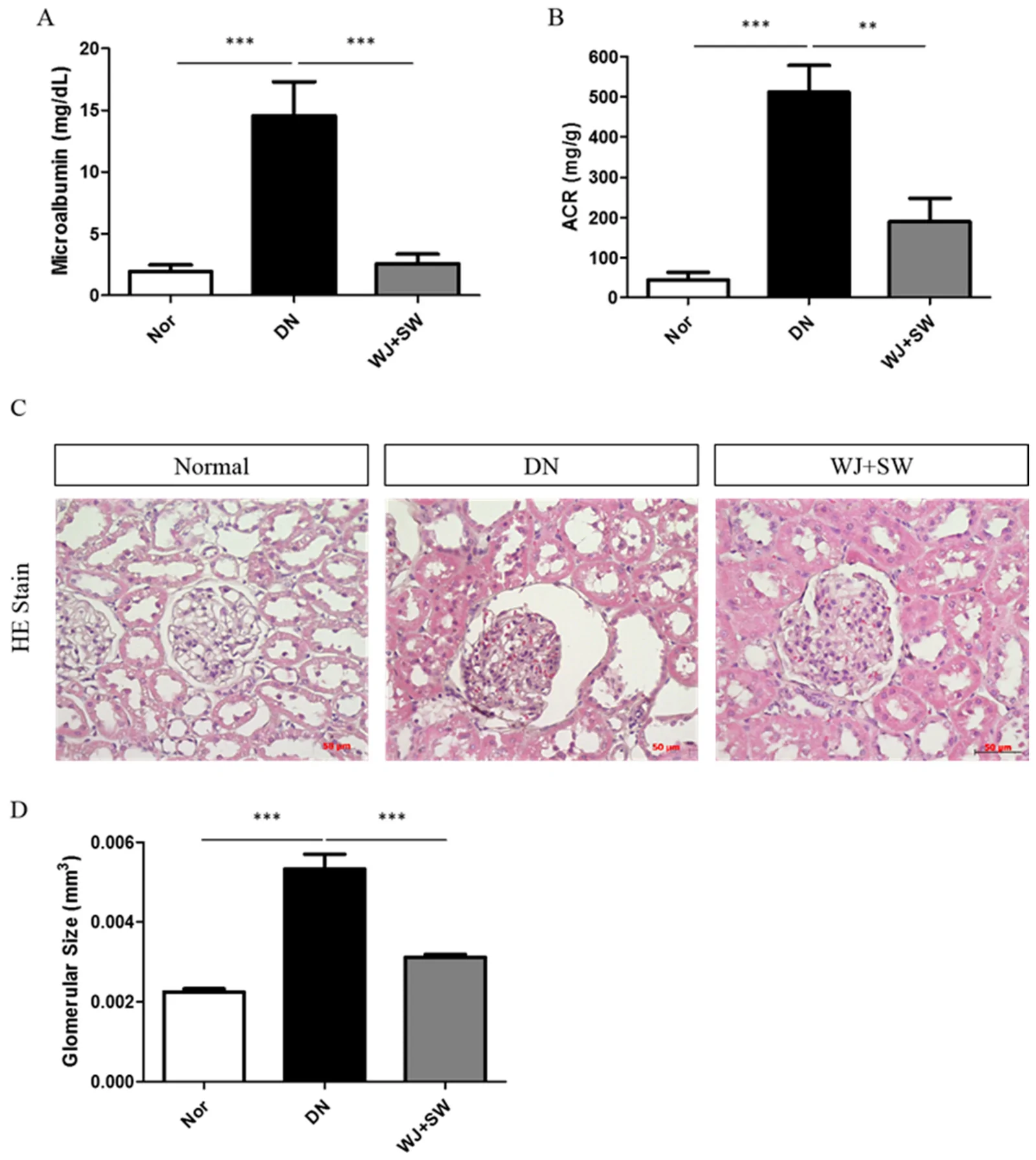
WJ + SW therapy improved kidney function in DN rats. (**A**) Microalbumin. *** *p* < 0.001 (N = 8). (**B**) Albumin-to-creatinine ratio (ACR). *** *p* < 0.001; ** *p* < 0.01 (N = 8). (**C**) Representative HE-stained renal tissue; bar = 50 μm. (**D**) Glomerular volume from HE-stained sections. *** *p* < 0.001 (N = 8). Nor, normal group; WJ + SW, low-energy SW + WJ-MSC therapy group; DN, diabetic nephropathy group.

HbA1C was measured at the end of the study (week 12). It was 4.3 ± 0.1% in the normal group, 10.7 ± 0.2% in the DN group, and 9.9 ± 0.3% in the intervention group (no difference between the DN and intervention groups, *p* < 0.05). The average blood sugar level in the DN group was 431.8 ± 15.9 mg/dL, and it was 376.7 ± 20.5 mg/dL in the WJ + SW group before intervention (week 6). All animals were sacrificed at week 12. Urinary microalbumin rose sharply in the DN group compared with normal controls (14.56 ± 2.74 vs. 1.95 ± 0.53 mg/dL), and WJ + SW therapy brought it down to 2.58 ± 0.77 mg/dL (Figure 2A). The albumin-to-creatinine ratio (ACR) showed the same pattern: 511.34 ± 66.90 mg/g in DN rats versus 44.41 ± 19.50 mg/g in controls, falling to 189.34 ± 58.29 mg/g after WJ + SW treatment (Figure 2B). Glomerular hypertrophy, a hallmark of DN [5], was also evident: glomerular volume was 0.005326 ± 0.00037 in DN rats compared with 0.002248 ± 0.000081 in controls, and the combined therapy reduced it to 0.003109 ± 0.000078 (Figure 2C,D). Glomerular architecture was preserved, and microalbuminuria was improved by WJ + SW therapy.

### 3.3. WJ + SW Therapy Attenuated Glomerular Fibrosis

**Figure 3:**
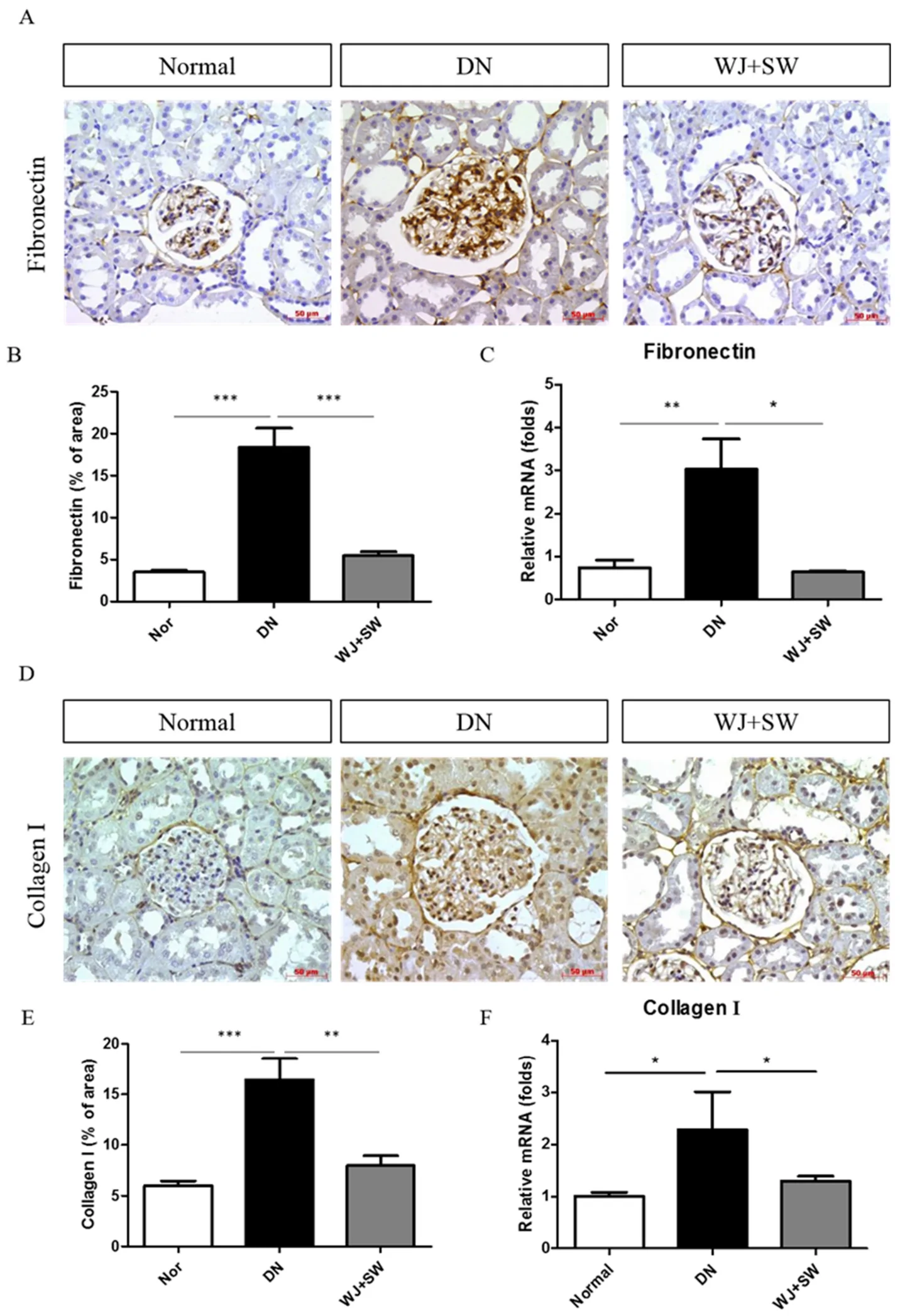
WJ + SW therapy significantly reduced glomerular fibrosis. (**A**,**B**,**D**,**E**) IHC staining for collagen I and fibronectin; bar = 50 μm. *** *p* < 0.001; ** *p* < 0.01 (N = 8). (**C**,**F**) PCR analyses of fibronectin and collagen I expression in kidney tissue. ** *p* < 0.01; * *p* < 0.05 (N = 8).

Fibrosis markers in the glomerulus were strongly elevated in DN rats and largely normalized by WJ + SW therapy. On IHC, fibronectin staining rose from 3.49 ± 0.20 in controls to 18.35 ± 2.28 in DN rats and fell to 5.45 ± 0.48 with treatment; collagen I went from 5.99 ± 0.47 to 16.25 ± 2.04 and back down to 9.29 ± 0.85. qPCR mirrored these changes: fibronectin transcripts rose from 0.74 ± 0.17 to 3.03 ± 0.70 and dropped to 0.64 ± 0.026 after WJ + SW treatment, while collagen I transcripts went from 1.00 ± 0.082 to 2.28 ± 0.84 and down to 0.54 ± 0.063 (Figure 3A–F). Both at the protein and transcript level, the combined therapy clearly blunted glomerular fibrogenesis.

### 3.4. WJ + SW Therapy Reduced Podocyte Injury and Improved Viability

**Figure 4:**
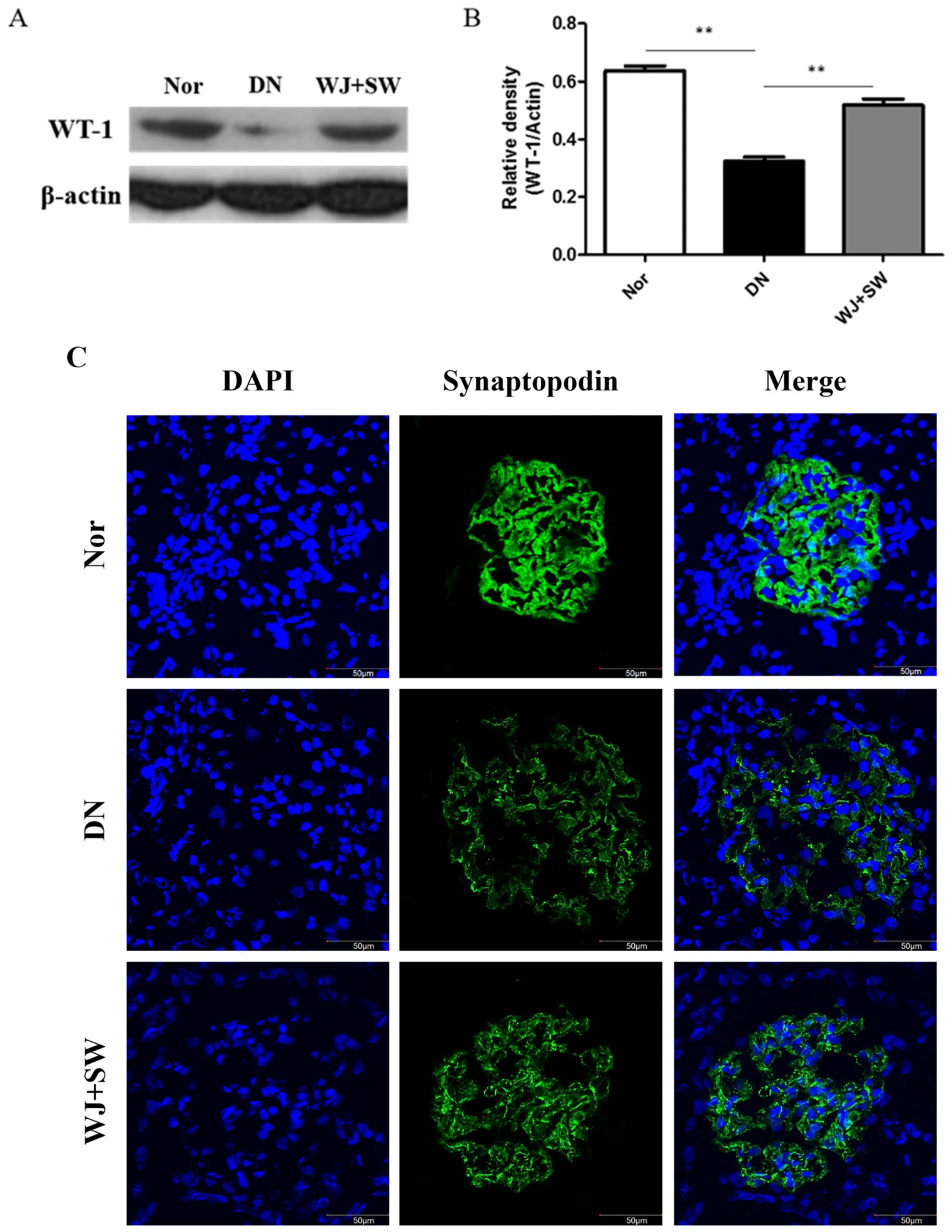

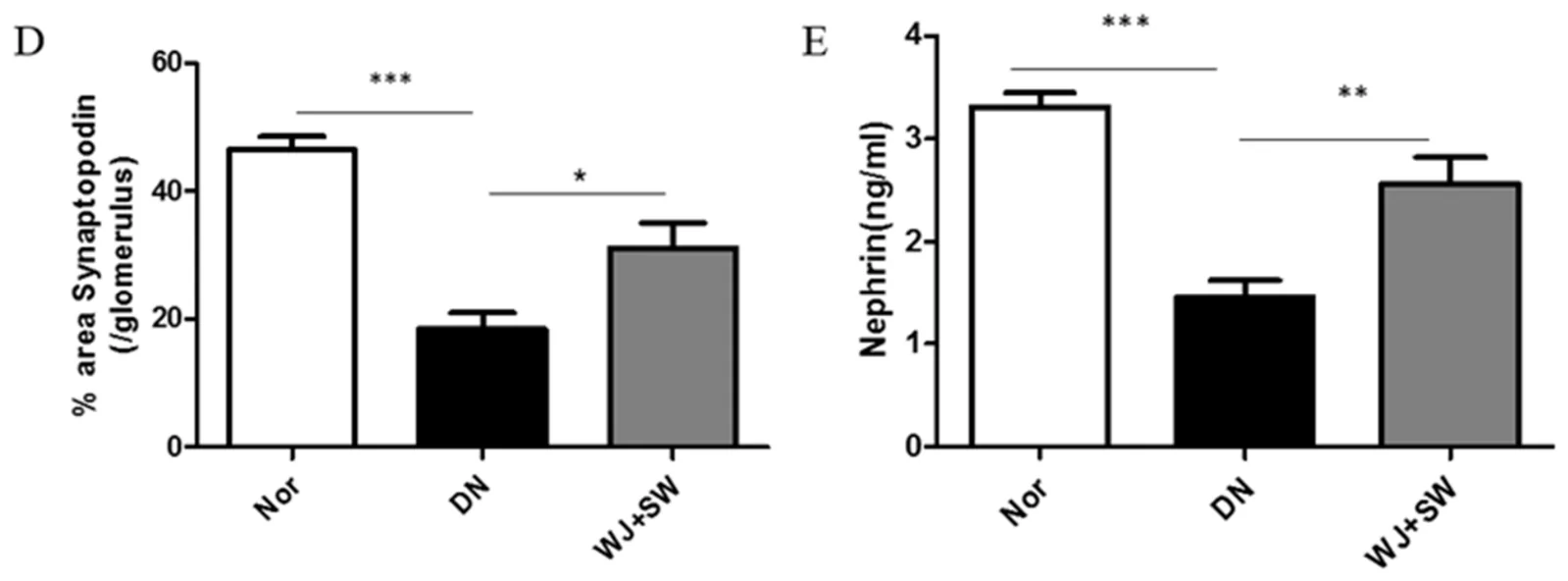
WJ + SW therapy significantly reduced podocyte injury and increased viability. (**A**,**B**) Western blotting for WT-1 with densitometric analysis. (**C**,**D**) IF staining for synaptopodin (green); bar = 50 μm. (**E**) ELISA for nephrin level in urine. *** *p* < 0.001; ** *p* < 0.01; * *p* < 0.05 (N = 8).

Podocyte markers were severely depleted in the DN kidney. The WT-1 signal fell from 0.64 ± 0.019 in controls to 0.32 ± 0.019 in DN rats, and synaptopodin dropped from 46.49 ± 2.11 to 18.43 ± 2.47. After WJ + SW therapy, both markers recovered toward control levels (WT-1 0.52 ± 0.024; synaptopodin 31.14 ± 3.96; Figure 4A–D). Urinary nephrin level was significantly lower in DN rats (1.46 ± 0.19 ng/mL) than in the control group (3.31 ± 0.16 ng/mL) and rose to 2.56 ± 0.29 ng/mL after treatment (Figure 4E).

### 3.5. WJ + SW Therapy Promoted Cell Proliferation, Reduced Apoptosis, and Enhanced Paracrine Activity

**Figure 5:**
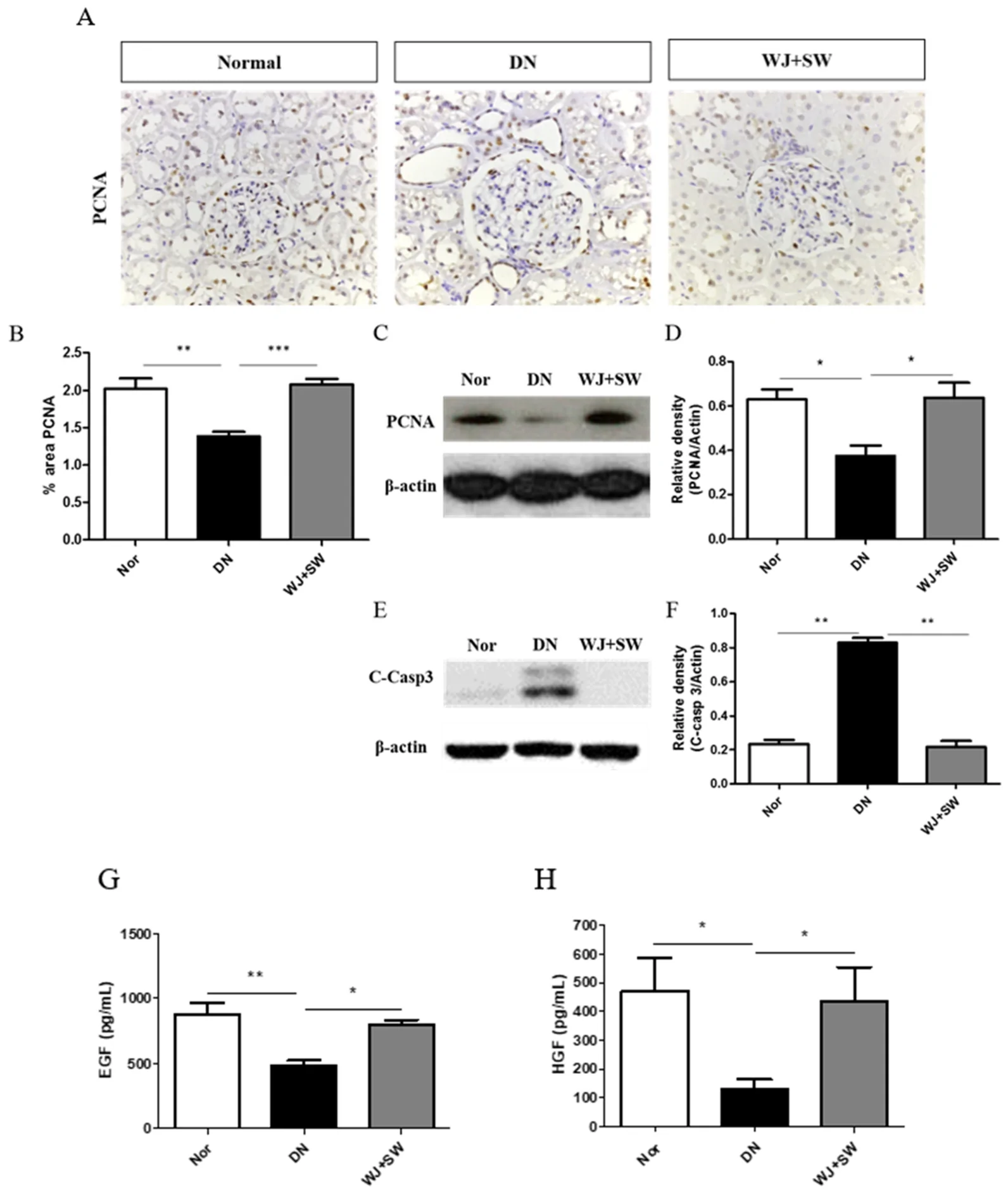
WJ + SW therapy significantly enhanced cell proliferation, decreased apoptosis, and increased paracrine activity. (**A**,**B**) IHC for PCNA; bar = 50 μm. (**C**,**D**) Western blotting for PCNA. (**E**,**F**) Western blotting for cleaved caspase-3. (**G**,**H**) ELISA for EGF and HGF levels (paracrine markers). *** *p* < 0.001; ** *p* < 0.01; * *p* < 0.05 (N = 8).

Cell turnover in the DN kidney was clearly disturbed. PCNA expression—an index of proliferation—dropped on IHC (1.38 ± 0.062 vs. 2.02 ± 0.14 in controls) and on Western blot (0.37 ± 0.055 vs. 0.63 ± 0.053). With WJ + SW therapy, both rose back to near-normal values (IHC 2.08 ± 0.072; WB 0.64 ± 0.078; Figure 5A–D). Apoptosis showed the opposite pattern: cleaved caspase-3 climbed from 0.23 ± 0.028 in controls to 0.83 ± 0.031 in DN rats and dropped to 0.22 ± 0.040 after treatment (Figure 5E,F). EGF and HGF—two paracrine factors—were also upregulated by WJ + SW therapy (EGF: 867.9 ± 99.8 pg/mL in normal group, 483.8 ± 38.5 pg/mL in DN and 799.9 ± 30.1 pg/mL in WJ + SW group; HGF: 471.2 ± 113.8 pg/mL in normal group, 127.4 ± 35.6 pg/mL in DN group and 435.8 ± 117.6 pg/mL in WJ + SW group, Figure 5G,H).

### 3.6. WJ + SW Therapy Suppressed Inflammation and Enhanced Anti-Inflammatory Activity

**Figure 6:**
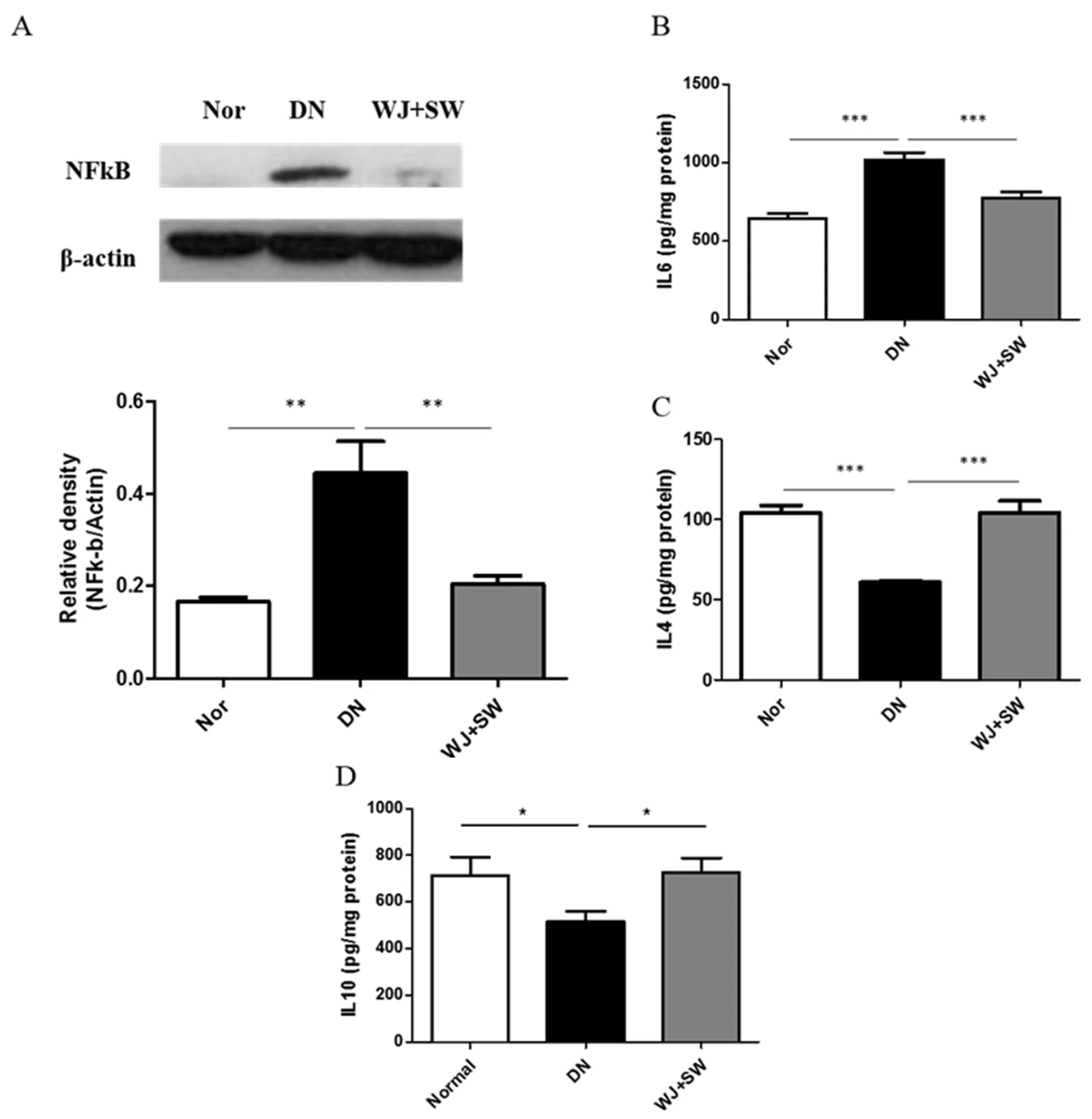
WJ + SW therapy significantly enhanced anti-inflammatory activity and decreased inflammation. (**A**) Western blotting for NF-κB. (**B**–**D**) ELISA for IL-4, IL-10, and IL-6. *** *p* < 0.001; ** *p* < 0.01; * *p* < 0.05 (N = 8).

NF-κB was markedly elevated in DN kidneys (0.44 ± 0.097 vs. 0.17 ± 0.013 in controls) and dropped to 0.20 ± 0.022 with WJ + SW therapy (Figure 6A). The same pattern held for IL-6, which rose to 1019.6 ± 45.3 pg/mg in DN rats from 644.8 ± 30.7 pg/mg in controls and fell to 774.4 ± 38.2 pg/mg after treatment (Figure 6D). Anti-inflammatory cytokines moved in the opposite direction: IL-4 declined in DN rats (60.7 ± 1.0 vs. 103.8 ± 4.7 pg/mg) and recovered to 104.2 ± 7.3 pg/mg with WJ + SW therapy; IL-10 followed the same trend (515.4 ± 43.9 vs. 713.7 ± 79.0 pg/mg in controls; 725.6 ± 63.4 pg/mg after treatment) (Figure 6B,C).

### 3.7. WJ + SW Therapy Polarized Macrophages from M1 to M2 Phenotype

**Figure 7:**
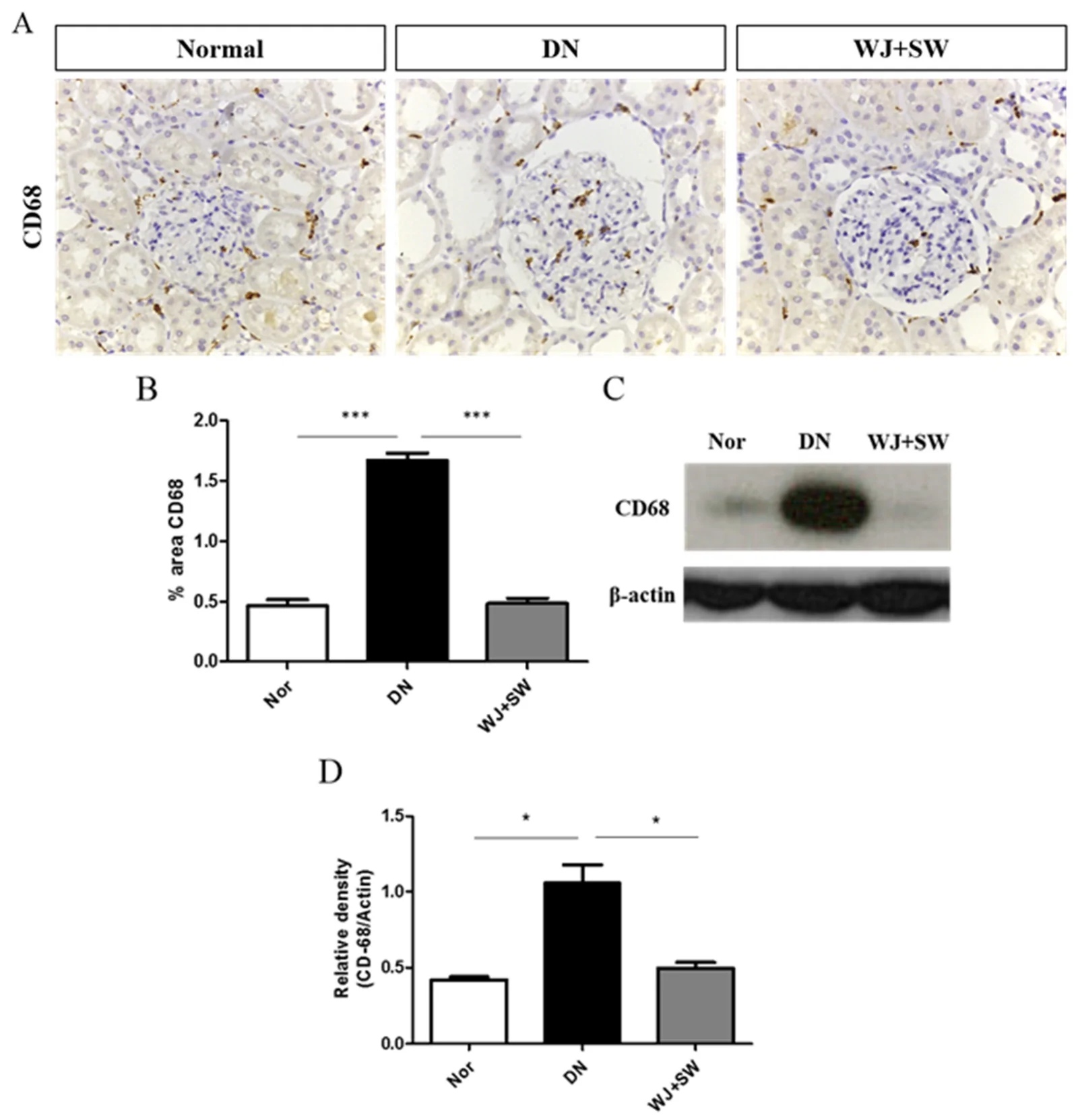

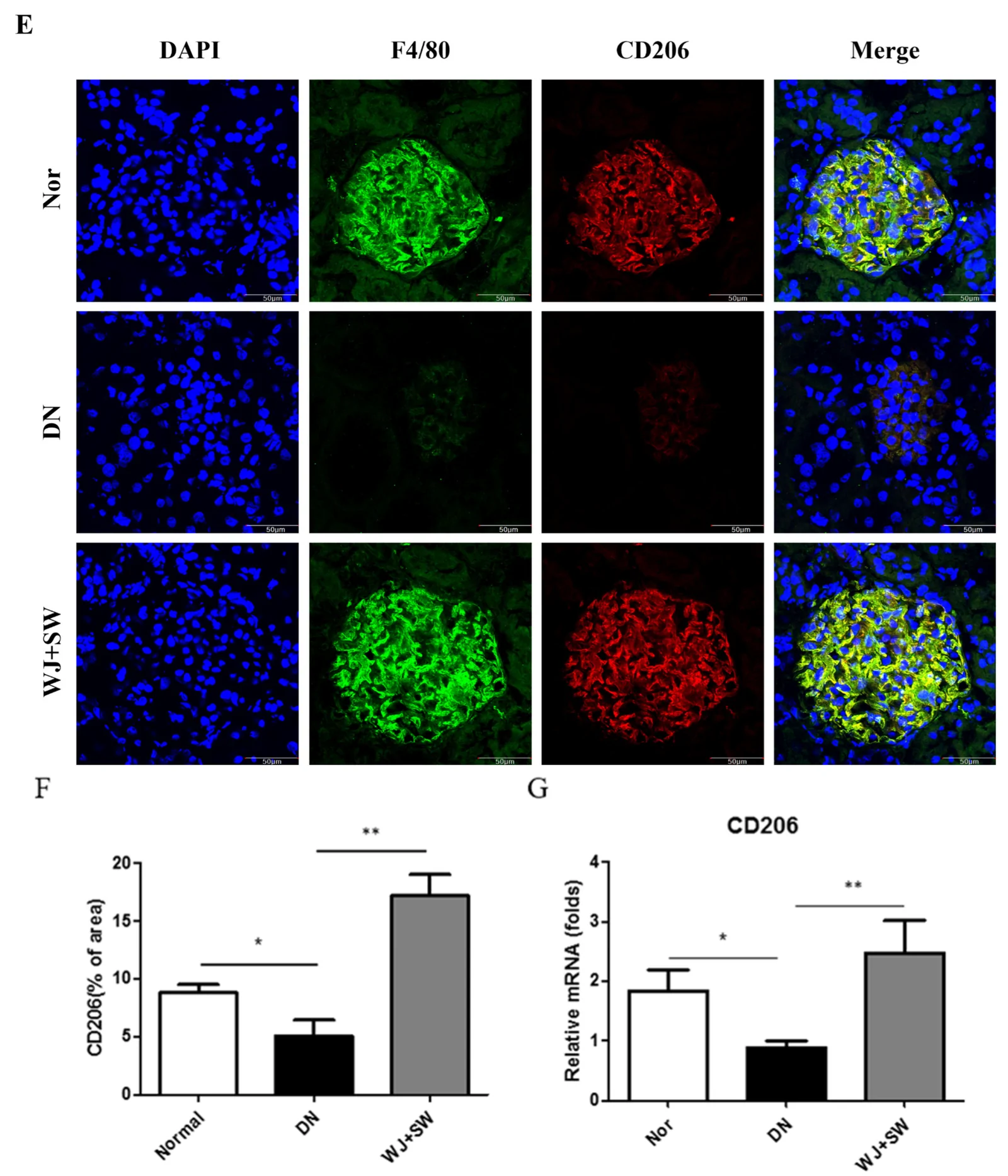
WJ + SW therapy significantly increased M2 macrophages and reduced M1 macrophages. (**A**,**B**) IHC for CD68 (M1 marker); bar = 50 μm. (**C**,**D**) Western blotting for CD68. (**E**,**F**) IF for CD206 (red) and F4/80 (green); bar = 50 μm. (**G**) PCR for CD206. *** *p* < 0.001; ** *p* < 0.01; * *p* < 0.05 (N = 8).

DN kidneys were heavily infiltrated by CD68^+^ M1 macrophages, with elevated readings on both IHC (1.67 ± 0.060 vs. 0.46 ± 0.053 in controls) and Western blot (1.055 ± 0.15 vs. 0.42 ± 0.025) (Figure 7A–D), and a parallel loss of CD206^+^ M2 macrophages on IF (5.02 ± 1.40 vs. 8.79 ± 0.68) and at the transcript level (CD206 mRNA 0.88 ± 0.12 vs. 1.84 ± 0.35). WJ + SW therapy reversed both arms of this shift: CD68^+^ cells fell to 0.48 ± 0.044 (IHC) and 0.50 ± 0.046 (WB), while CD206^+^ M2 cells rose to 17.15 ± 1.83 (IF) and CD206 transcripts to 2.47 ± 0.55 (Figure 7E–G). The treatment thus pushed the renal macrophage profile from a pro-inflammatory M1 dominance toward an M2-rich, reparative state [29].

### 3.8. WJ + SW Therapy Reduced Oxidative Stress and Enhanced Antioxidative Defense

**Figure 8:**
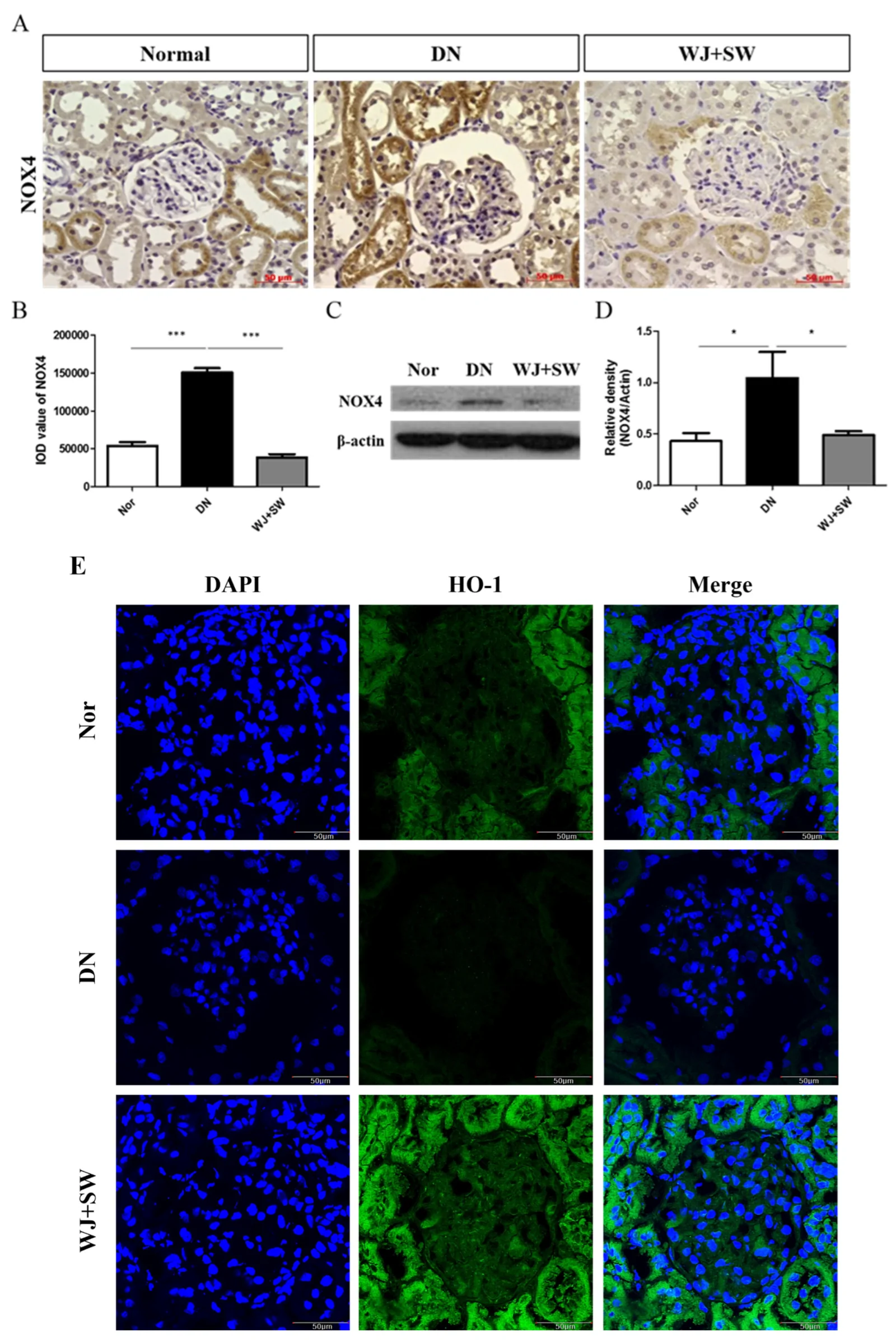

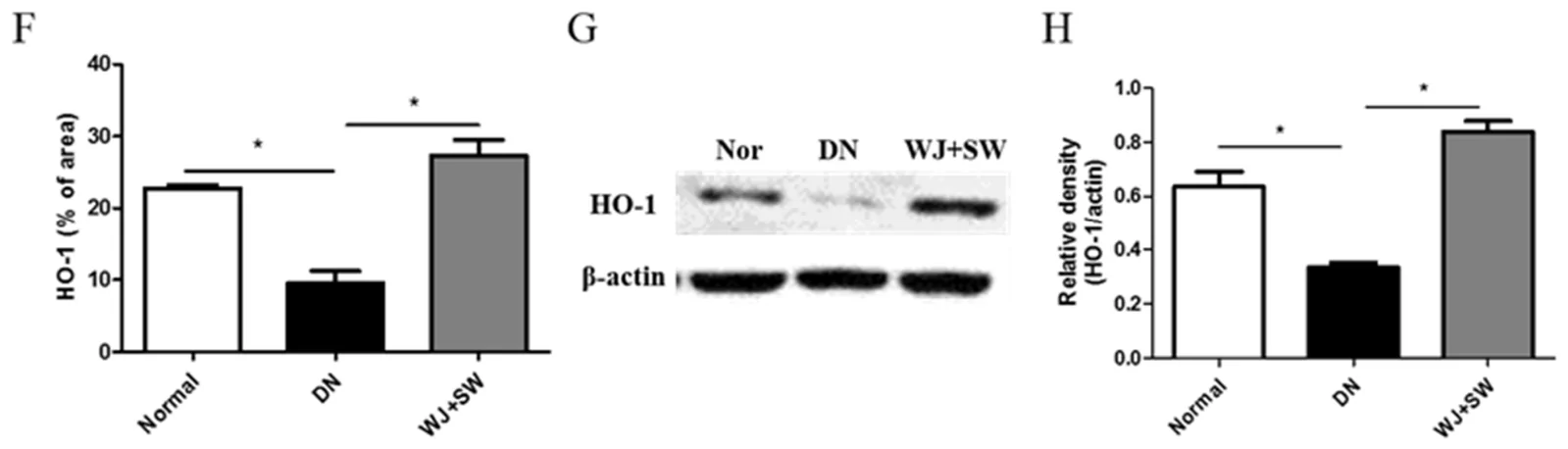
WJ + SW therapy significantly increased antioxidative defense and reduced oxidative stress. (**A**,**B**) IHC for NOX4; bar = 50 μm. (**C**,**D**) Western blotting for NOX4. (**E**,**F**) IF for HO-1; bar = 50 μm. (**G**,**H**) Western blotting for HO-1. *** *p* < 0.001; * *p* < 0.05 (N = 8).

NOX4, the dominant NADPH oxidase in diabetic kidneys, was strongly induced in DN rats on both IHC (150,735.37 ± 5812.65 vs. 53,864.41 ± 4865.94 in controls) and Western blot (1.045 ± 0.31 vs. 0.43 ± 0.093), and WJ + SW therapy brought it back down (IHC 38,526.18 ± 4370; WB 0.49 ± 0.048) (Figure 8A–D). HO-1, a key cytoprotective antioxidant, moved in the opposite direction: down in DN (IF 9.50 ± 2.44 vs. 22.78 ± 0.64; WB 0.33 ± 0.021 vs. 0.64 ± 0.067) and up after treatment (IF 27.30 ± 2.71; WB 0.84 ± 0.050) (Figure 8E–H).

## 4. Discussion

Our data show that combining low-energy shock wave therapy with WJ-MSCs produces a potential therapeutic effect in a streptozotocin-induced rat model of DN. The combined regimen reduced microalbuminuria, ACR, glomerular fibrosis and hypertrophy; restored podocyte markers, nephrin expression, and paracrine activity; suppressed inflammation and oxidative stress; and tilted renal macrophages from M1 to M2. Taken together, the results support WJ + SW therapy as a multi-target candidate for DN.

DN arises from a tangled interplay between hyperglycemia, chronic inflammation, oxidative stress, and apoptosis [5,6]. Recent integrative work has pinpointed NF-κB, IL-6, and TNF-α as central nodes of immune-metabolic injury in the diabetic kidney [1,6]. In our rats, WJ + SW therapy lowered renal NF-κB and IL-6 and raised IL-4 and IL-10—an anti-inflammatory profile consistent with earlier work showing that low-energy SW therapy reduces inflammation in DN [15,27] and in diabetes mellitus more broadly [25]. It has been reported that WJ-MSCs exhibit anti-inflammatory properties [18] and can even blunt sepsis-induced organ injury through cholinergic anti-inflammatory pathways [21]. Our results indicated that this combination tilts the renal cytokine milieu away from inflammation.

One of the more striking findings was the robust shift from CD68^+^ M1 to CD206^+^ M2 macrophages within the diabetic kidney. Macrophage polarization is now considered a central driver of DN: sustained M1 dominance fuels inflammation and fibrosis, while M2 polarization helps resolve injury and supports repair [14,30]. Targeting this balance has emerged as a promising therapeutic strategy in DN [31]. Umbilical cord-derived MSCs have already been shown to reprogram renal macrophages toward M2 and protect the diabetic kidney [32], and low-intensity ESWT reduces renal inflammation and fibrosis without nephrotoxicity in both animal and human studies [27]. Combining the two treatments may amplify M1-to-M2 polarization beyond what either modality has been reported to achieve on its own. Further study is required to clarify the mechanism.

Oxidative stress, much of it driven by NOX4, contributes substantially to inflammation, podocyte apoptosis, and tubular injury in DN [8,9]. NOX4 deletion attenuates albuminuria, glomerulosclerosis, and ECM accumulation in experimental models [8]. In our rats, WJ + SW therapy strongly suppressed renal NOX4 and elevated HO-1, a key cytoprotective antioxidant. These changes line up with prior reports that SW therapy reduces oxidative stress in DN and diabetes [15,25] and that WJ-MSCs deliver antioxidant effects largely through paracrine mechanisms [20,22]. The combined therapy thus dampened oxidative stress while reinforcing antioxidant defenses, in line with oxidative-stress biomarkers relevant to DN progression [33].

Podocyte loss is widely regarded as the central event driving proteinuria and glomerulosclerosis in DN [10,11]. Nephrin maintains the slit-diaphragm and barrier function, and urinary nephrin has emerged as an early biomarker of the disease [11]. Moreover, WT-1 and synaptopodin are both widely used as sensitive indicators of glomerular injury and recovery in diabetic nephropathy [34]. In our experiments, animals with DN showed a marked reduction of nephrin (50%), indicating podocyte injury and/or loss. This decrease rebounded after WJ + SW therapy. In association with increased WT-1 and synaptopodin, our results suggested that combined therapy diminished podocyte injury and restored slit diaphragm integrity. The parallel rise in PCNA-positive proliferating cells and drop in cleaved caspase-3 imply the treatment promotes cell proliferation while curbing apoptotic loss. Increased HGF and EGF levels also point to paracrine signaling, a recognized mechanism by which WJ-MSCs drive tissue repair [20,23].

The effects obtained in WJ + SW therapy make mechanistic sense. Shock waves deliver mechanotransduction signals that turn on endogenous regenerative pathways and produce anti-inflammatory and anti-oxidative responses [15,25,27]. WJ-MSCs, in turn, supply paracrine factors—HGF and EGF chief among them—that directly alleviate podocyte injury and improve survival [20,23] and modulate innate and adaptive immunity [18,19,24]. By acting on different pathogenic axes at once, the combination may amplify treatment effects, consistent with recent calls for multi-target precision interventions in DN [1,5].

This study has several limitations. Firstly, our study investigated the effects of combined treatments, and monotherapy was not included. Because individual therapy has already been shown to have its therapeutic potential in DN, we aimed to examine whether combination therapy can be a harmless and technically potential treatment modality. Second, although we identified several key downstream effectors (NOX4, HO-1, IL-6, IL-10, NF-κB, nephrin), the exact molecular bridge between SW-induced mechanotransduction and WJ-MSC paracrine signaling is still unclear. A bi-directional reaction between stem cell therapy and SW has been proposed, and therapeutic effects have been demonstrated in several disease models, including chronic kidney disease [35]. Third, special staining such as PAS staining or Sirius red staining is helpful to correlate our results in the present study and provide alterations in glomerular structure more precisely. Future studies are mandatory to prove this relationship. Fourth, long-term safety, optimal dosing, probable additive effects from preparation solutions such as hyaluronic acid, and durability of effects were also not assessed and warrant further investigation. Finally, WJ-MSC-derived exosomes or extracellular vesicles retain much of the parent cell’s paracrine activity in a cell-free format [23] and have been shown to drive M2 polarization and protect the diabetic kidney [36]; they may offer a more translationally tractable form of this combined approach in future work.

## 5. Conclusions

Combining low-energy SW therapy with WJ-MSCs reduced urinary microalbumin, glomerular hypertrophy, and renal fibrosis in diabetic rats. Shock wave therapy acted through mechanotransduction—blunting oxidative stress, inflammation, and apoptosis while supporting cell proliferation and repair. WJ-MSCs added paracrine support, releasing HGF and EGF that reduced podocyte injury. Together, the two approaches produced a multi-target effect that meaningfully alleviated DN in this model. The exact molecular wiring of the synergy will require further work to define.

## Data Availability

All data generated or analyzed during this study are included in this published article.
